# Prognostic significance of the combination of preoperative hemoglobin, albumin, lymphocyte and platelet in patients with gastric carcinoma: a retrospective cohort study

**DOI:** 10.18632/oncotarget.5629

**Published:** 2015-10-15

**Authors:** Xiao-Long Chen, Lian Xue, Wei Wang, Hai-Ning Chen, Wei-Han Zhang, Kai Liu, Xin-Zu Chen, Kun Yang, Bo Zhang, Zhi-Xin Chen, Jia-Ping Chen, Zong-Guang Zhou, Jian-Kun Hu

**Affiliations:** ^1^ Department of Gastrointestinal Surgery, West China Hospital, Sichuan University, Chengdu, Sichuan, China; ^2^ Laboratory of Gastric Cancer, State Key Laboratory of Biotherapy, West China Hospital, Sichuan University, Chengdu, Sichuan, China; ^3^ Laboratory of Digestive Surgery, State Key Laboratory of Biotherapy, West China Hospital, Sichuan University, Chengdu, Sichuan, China

**Keywords:** gastric carcinoma, hemoglobin, albumin, lymphocyte, platelet

## Abstract

Nutritional and immune status is important to the prognosis of patients with gastric carcinoma (GC). Here, we evaluated the prognostic significance of the combination of preoperative hemoglobin, albumin, lymphocyte and platelet (HALP) in patients with GC. From January 2005 to December 2011, 1332 patients with GC who underwent gastrectomy were randomly divided into the training (*n* = 888) and the validation sets (*n* = 444) by X-tile according to the sample size ratio 2:1. The cut-point of HALP was 56.8 and the patients were subsequently subdivided into HALP < 56.8 and HALP ≥ 56.8 groups in both two sets. Multivariate analysis revealed that gender (*p* < 0.001, *p* < 0.001), tumor size (*p* = 0.003, *p* = 0.035) and T stage (*p* < 0.001, *p* = 0.044) were independently related to HALP both in the training and the validation sets. Kaplan-Meier (*p* < 0.001, *p* = 0.003) and Cox regression (*p* = 0.043, *p* = 0.042) showed that the prognosis of HALP ≥ 56.8 group was significantly better than that of HALP < 56.8 group both in two sets (*p* < 0.001, *p* < 0.001). Nomograms of these two sets based on HALP was more accurate in prognostic prediction than TNM stage alone. Our findings suggested that HALP was closely associated with clinicopathological features and was an independent prognostic factor in GC patients. Nomogram based on HALP could accurately predict the prognosis of GC patients.

## INTRODUCTION

Gastric carcinoma (GC) is one of the most common malignances in the world, especially in East Asia [1]. Surgery is still the main treatment for resectable GC. Neoadjuvant and adjuvant chemotherapy is demonstrated to be valuable to improve the prognosis of patients [2]. However, because of recurrence and metastasis, the mortality of GC is still high. At present, TNM stage involving invasion depth, metastasis of lymph nodes and distant metastasis of tumor has been considered as the primary factor to predict the prognosis [1, 3]. Whereas, TNM stage only reflects the characteristics of cancer itself. Notably, the outcomes of some patients with the same stage might be completely different. Therefore, it is crucially important to find out other ways to increase the predictive accuracy of the prognosis in GC patients.

The prognosis of patients with cancer is the integrated outcome between tumor aggression and body defense. Immune, as the main resource of the defense against cancer, has gradually become the focus in the field of cancer research nowadays. Immunotherapy has also manifested as a potential treatment for some selected cancers, like melanoma [4]. The nutritional status of patients with cancer is another important parameter affecting survival outcomes, especially in the older patients with chemotherapy [5]. As the most common preoperative examinations, the results of preoperative complete blood count and liver function tests have been studied as the promising prognostic predictors in some tumors, like hepatocellular carcinoma and GC [6].

Platelet count was found increasing in lung cancer and colorectal cancer, which indicated poor survival outcomes [7]. Lymphocyte plays an important role in the defense against cancer through inducing cytotoxic cell death and inhibiting proliferation and migration of cancer cell [8]. Hemoglobin and albumin are two of the most common indexes to reflect the performance and nutritional status of patients. Hemoglobin has been reported as a prognostic factor in cancer patients, and anemia was associated with poor prognosis [9]. Albumin has also been demonstrated as a prognostic factor in GC, revealing that patients with higher level of albumin had better prognosis than those with lower level of albumin [10].

Among these indexes, platelets to lymphocytes ratio (PLR) and albumin multiplying lymphocytes known as the prognostic nutritional index (PNI) have been extensively studied in GC [11, 12]. These reports showed that PLR and PNI seemed promising to predict the prognosis, but without internal or external validation cohort to enhance the conclusion. Meanwhile, hemoglobin, albumin and lymphocyte may be positive correlated with prognosis, but platelet may be negative. Therefore, the combination of these four indexes seems feasible and reasonable in prediction of prognosis. To our best knowledge, no studies had investigated the significance of the combination of hemoglobin, albumin, lymphocyte and platelet in GC. Hereby, the aim of this study was to research the clinical value of the combination of these four indexes in GC patients.

## RESULTS

### The relationship between the combination of hemoglobin, albumin, lymphocytes and platelets (HALP) and clinicopathological features

In this study, 888 (66.7%) patients were enrolled into the training set (death *n* = 388), with 618 patients in low HALP (LHALP) group (death *n* = 296) and 270 patients in high HALP (HHALP) group (death *n* = 92). And there were 444 (33.3%) patients in the validation set (death *n* = 193), with 309 patients in LHALP group (death *n* = 148) and 135 patients in HHALP group (death *n* = 45). We first compared the clinicopathological characteristics between the training and the validation sets. And the result showed that the differences in all these features between the training set and the validation set were not significant, except longitudinal location (*p* = 0.039) (Table 1), indicating the similar constitution and comparability between the training and the validation sets.

**Table 1:** The clinicopathological features of patients in the training set and the validation set in this study

			Training	set			Validation	set		
Clinicopathological	features	HALP < 56.8	HALP ≥ 56.8	*P*	Total	HALP < 56.8	HALP ≥ 56.8	*P*	Total	*P*#
		*n* = 618 (%/SD)	*n* = 270 (%/SD)	value	*n* = 888 (%/SD)	*n* = 309 (%/SD)	*n* = 135 (%/SD)	value	*n* = 444 (%/SD)	value
Age (years)	Mean ± SD	58.1 ± 11.8	55.6 ± 11.7	0.005	57.3 ± 11.8	57.3 ± 11.8	55.6 ± 10.8	0.157	56.8 ± 11.5	0.422
	≥ 60	299 (48.4)	115 (42.6)	0.112	414 (46.6)	131 (42.4)	52 (38.5)	0.445	183 (41.2)	0.061
	< 60	319 (51.6)	155 (57.4)		474 (53.4)	178 (57.6)	83 (61.5)		261 (58.8)	
Gender	Male	418 (67.6)	221 (81.9)	<0.001	639 (72.0)	196 (63.4)	108 (80.0)	<0.001	304 (68.5)	0.187
	Female	200 (32.4)	49 (18.1)		249 (28.0)	113 (36.6)	27 (20.0)		140 (31.5)	
Longitudinal	U	146 (23.6)	47 (17.4)	0.118	193 (21.7)	64 (20.7)	27 (20.0)	0.771	91 (20.5)	0.039
location	M	132 (21.4)	53 (19.6)		185 (20.8)	68 (22.0)	27 (20.0)		95 (21.4)	
	L	333 (53.9)	167 (61.9)		500 (56.3)	165 (53.4)	78 (57.8)		243 (54.7)	
	UML	7 (1.1)	3 (1.1)		10 (1.1)	12 (3.9)	3 (2.2)		15 (3.4)	
Cross sectional	Lesser curvature	337 (54.5)	147 (54.4)	0.571	484 (54.5)	156 (50.5)	80 (59.3)	0.343	236 (53.2)	0.575
location	Greater curvature	52 (8.4)	28 (10.4)		80 (9.0)	30 (9.7)	14 (10.4)		44 (9.9)	
	Anterior wall	37 (6.0)	13 (4.8)		50 (5.6)	18 (5.8)	10 (7.4)		28 (6.3)	
	Posterior wall	58 (9.4)	23 (8.5)		81 (9.1)	24 (7.8)	8 (5.9)		32 (7.2)	
	Double walls	52 (8.4)	30 (11.1)		82 (9.2)	40 (12.9)	12 (8.9)		52 (11.7)	
	All walls	82 (13.3)	29 (10.7)		111 (12.5)	41 (13.3)	11 (8.1)		52 (11.7)	
Macroscopic	0-II	383 (62.0)	196 (72.6)	0.002	579 (65.2)	201 (65.0)	99 (73.3)	0.086	300 (67.6)	0.390
type	III-IV	235 (38.0)	74 (27.4)		309 (34.8)	108 (35.0)	36 (26.7)		144 (32.4)	
Differentiation	Well/Moderately	118 (19.1)	61 (22.6)	0.232	179 (20.2)	63 (20.4)	30 (22.2)	0.663	93 (20.9)	0.737
grade	Poorly	500 (80.9)	209 (77.4)		709 (79.8)	246 (79.6)	105 (77.8)		351 (79.1)	
Tumor size	Mean ± SD	5.2 ± 2.6	4.11 ± 2.7	<0.001	4.9 ± 2.6	5.4 ± 3.3	3.9 ± 2.1	<0.001$	4.9 ± 3.1	0.783$
(cm)	< 5	277 (44.8)	171 (63.3)	<0.001	448 (50.5)	137 (44.3)	85 (63.0)	<0.001	222 (50.0)	0.877
	≥ 5	341 (55.2)	99 (36.7)		440 (49.5)	172 (55.7)	50 (37.0)		222 (50.0)	
Vessels/nerves	Negative	482 (78.0)	227 (84.1)	0.038	709 (79.8)	247 (79.9)	118 (87.4)	0.059	365 (82.2)	0.303
invasion	Positive	136 (22.0)	43 (15.9)		179 (20.2)	62 (20.1)	17 (12.6)		79 (17.8)	
T stage	1–2	182 (29.4)	134 (49.6)	<0.001	316 (35.6)	96 (31.1)	68 (50.4)	<0.001	164 (36.9)	0.628
	3–4	436 (70.6)	136 (50.4)		572 (64.4)	213 (68.9)	67 (49.6)		280 (63.1)	
N stage	0	180 (29.1)	113 (41.9)	<0.001	293 (33.0)	97 (31.4)	62 (45.9)	0.002	159 (35.8)	0.184
	1	94 (15.2)	39 (14.4)		133 (15.0)	62 (20.1)	24 (17.8)		86 (19.4)	
	2	123 (19.9)	43 (15.9)		166 (18.7)	43 (13.9)	14 (10.4)		57 (12.8)	
	3a	141 (22.8)	51 (18.9)		192 (21.6)	63 (20.4)	27 (20.0)		90 (20.3)	
	3b	80 (12.9)	24 (8.9)		104 (11.7)	44 (14.2)	8 (5.9)		52 (11.7)	
M stage	0	580 (93.9)	265 (98.1)	0.006	845 (95.2)	282 (91.3)	130 (96.3)	0.059	412 (92.8)	0.078
	1	38 (6.1)	5 (1.9)		43 (4.8)	27 (8.7)	5 (3.7)		32 (7.2)	
TNM stage	IA	79 (12.8)	81 (30.0)	<0.001	160 (18.0)	40 (12.9)	38 (28.1)	<0.001	78 (17.6)	0.507
	IB	49 (7.9)	23 (8.5)		72 (8.1)	37 (12.0)	13 (9.6)		50 (11.3)	
	IIA	36 (5.8)	17 (6.3)		53 (6.0)	23 (7.4)	10 (7.4)		33 (7.4)	
	IIB	81 (13.1)	27 (10.0)		108 (12.2)	36 (11.7)	21 (15.6)		57 (12.8)	
	IIIA	74 (12.0)	36 (13.3)		110 (12.4)	31 (10.0)	13 (9.6)		44 (9.9)	
	IIIB	94 (15.2)	25 (9.3)		119 (13.4)	39 (12.6)	11 (8.1)		50 (11.3)	
	IIIC	167 (27.0)	56 (20.7)		223 (25.1)	76 (24.6)	24 (17.8)		100 (22.5)	
	IV	38 (6.1)	5 (1.9)		43 (4.8)	27 (8.7)	5 (3.7)		32 (7.2)	

Abbreviations: HALP: Hemoglobin*Albumin*Lymphocyte/Platelet index; P# value: the difference between the training set and the validation set; SD: standard deviation; $: rank sum test.

In the training set, the univariate analysis showed that there were significantly more patients with younger age, male, macroscopic type 0-II, smaller tumor size, negative vessels/nerves invasion, T1-T2 stage, N0 stage, M0 stage, and TNM IA stage in HHALP group than those in LHALP group (all *p* < 0.05). Similarly, in the validation set, the patients in HHALP group had significantly more male, smaller tumor size, T1-T2 stage, N0 stage, and TNM IA stage (all *p* < 0.05). The multivariate analysis revealed that gender (*p* < 0.001), tumor size (*p* = 0.003) and T stage (*p* < 0.001) in the training set, and gender (*p* < 0.001), cross-sectional location (*p* = 0.032), tumor size (*p* = 0.035) and T stage (*p* = 0.044) in the validation set were independently related to HALP (Table 2).

**Table 2:** Multivariate logistic regression analyses of the relationship between Hemoglobin*Albumin*Lymphocyte/Platelet index with clinicopathological features in the training set and the validation set in this study

Clinicopathological features	Training	set (*n* = 888)	Validation	set (*n* = 444)
	*P* value	EXP (95% CI)	*P* value	EXP (95% CI)
Gender	<0.001	2.495 (1.727, 3.605)	<0.001	2.526 (1.537, 4.150)
Tumor size	0.003	0.779 (0.659, 0.920)	0.035	0.766 (0.597, 0.982)
T stage	<0.001	0.811 (0.724, 0.907)	0.044	0.844 (0.716, 0.995)
Cross-sectional location	−	−	0.032	0.880 (0.783, 0.989)

Abbreviations: CI: confidence interval.

### Prognostic significance of HALP

The median survival time and the 1, 2, 3-year overall survival rates of LHALP and HHALP groups in the training set and the validation set were shown in Table 3, indicating that HHALP group had longer median survival time and higher 1, 2, 3-year overall survival rates than LHALP group both in the two sets.

**Table 3:** Median survival time and 1, 2, 3-year overall survival rates of patients in the training set and the validation set in this study

	Training	set (*n* = 888)	Validation	set (*n* = 444)
	HALP < 56.8 (*n* = 618)	HALP ≥ 56.8 (*n* = 270)	HALP < 56.8 (*n* = 309)	HALP ≥ 56.8 (*n* = 135)
Median survival time (months)	67.7 (0.3–116.2)	108.0 (0.6–118.0)	68.8 (0.9–115.7)	108.0 (3.9–115.1)
1-year overall survival rates (%)	78.0	83.7	76.7	85.2
2-year overall survival rates (%)	67.1	78.8	66.9	79.2
3-year overall survival rates (%)	59.7	74.7	57.5	73.5

Abbreviations: HALP: Hemoglobin*Albumin*Lymphocyte/Platelet index.

**Figure 1:**
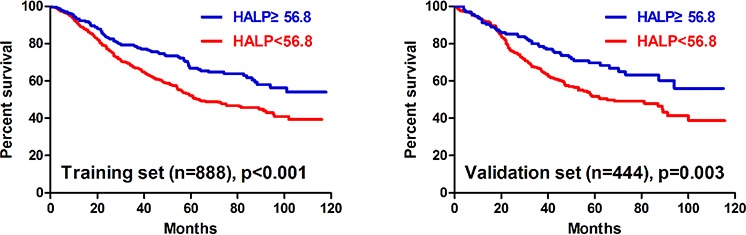
Survival analysis of HALP in the training and the validation sets

**Figure 2:**
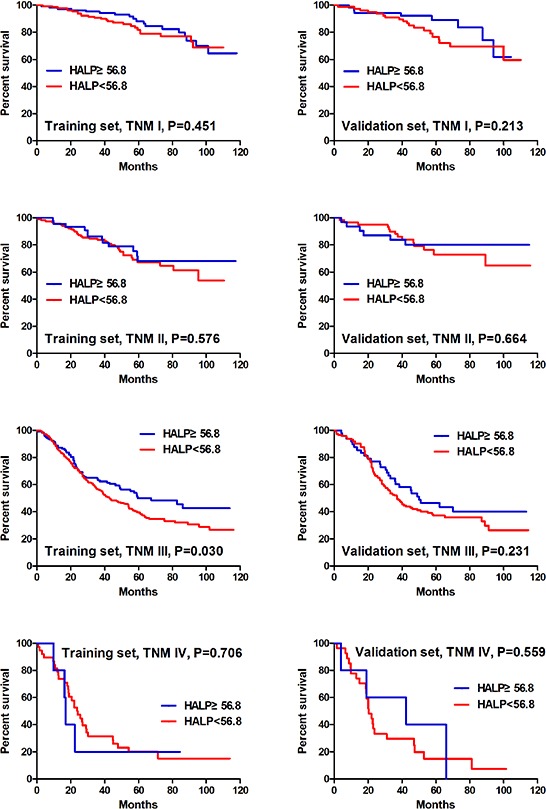
Survival analysis of HALP stratified by TNM stage in the training and the validation sets

Univariate analyses showed that all clinicopathological features (*p* < 0.05) but gender (*p* = 0.085) were significantly related to the survival outcomes in the training set. In the validation set, all clinicopathological characteristics (*p* < 0.05) except age (*p* = 0.074) and gender (*p* = 0.222) were obviously associated with prognosis. HHALP group had significantly better prognosis than LHALP group both in the training set (*p* < 0.001) and the validation set (*p* = 0.003) (Figure 1). Furtherly, we analyzed the prognostic significance of HALP stratified by TNM stage. The results revealed that HHALP group had remarkably better survival outcome than LHALP group only in the patients with TNM III stage in the training set (*p* = 0.030). However, no similar results were found in other subgroups both in the training and the validation sets (Figure 2).

In multivariate analyses, the results revealed that age (*p* = 0.002), longitudinal location (*p* = 0.004), tumor size (*p* = 0.001), N stage (*p* < 0.001), M stage (*p* = 0.045) and HALP (*p* = 0.043) were independent prognostic factors in the training set. And in the validation set, age (*p* = 0.045), longitudinal location (*p* = 0.017), N stage (*p* < 0.001) and HALP (*p* = 0.042) were independently associated with prognosis. Both in the training and the validation sets, HALP was confirmed as an independent prognostic factor (Table 4).

**Table 4:** Univariate and multivariate Cox regression analyses in the prognosis in the training set and the validation set in this study

Clinicopathological	Training set (*n* = 888)			Validation set (*n* = 444)		
features	Univariate	Multivariate		Univariate	Multivariate	
	*P* value	Hazard ratio (95% CI)	*P* value	*P* value	Hazard ratio (95% CI)	*P* value
Age	0.001	1.363 (1.116, 1.665)	0.002	0.074	1.349 (1.007, 1.806)	0.045
Gender	0.085	–	–	0.222	–	–
Longitudinal location	<0.001	1.071 (1.022, 1.123)	0.004	<0.001	1.084 (1.015, 1.159)	0.017
Cross sectional location	<0.001	–	–	<0.001	–	–
Macroscopic type	<0.001	–	–	<0.001	–	–
Differentiation grade	0.004	–	–	0.015	–	–
Tumor size	<0.001	1.199 (1.073, 1.341)	0.001	<0.001	–	–
Vessels/nerves invasion	<0.001	–	–	0.002	–	–
T stage	<0.001	–	–	<0.001	–	–
N stage	<0.001	1.631 (1.501, 1.772)	<0.001	<0.001	1.649 (1.488, 1.828)	<0.001
M stage	<0.001	1.449 (1.009, 2.082)	0.045	<0.001	–	–
TNM stage	<0.001	–	–	<0.001	–	–
HALP	<0.001	0.782 (0.617, 0.993)	0.043	0.003	0.700 (0.496, 0.987)	0.042

Abbreviations: HALP: Hemoglobin*Albumin*Lymphocyte/Platelet index; CI: confidence interval.

### Nomogram of the training set and the validation set

**Figure 3:**
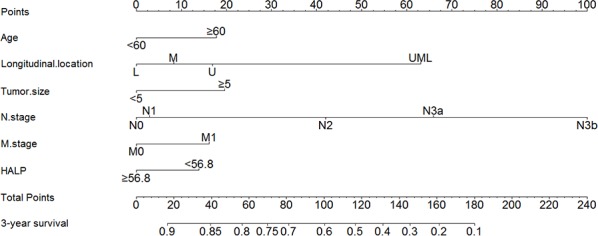
Nomogram of the training set

**Figure 4:**
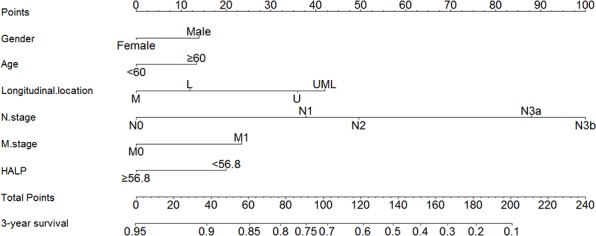
Nomogram of the validation set

**Figure 5:**
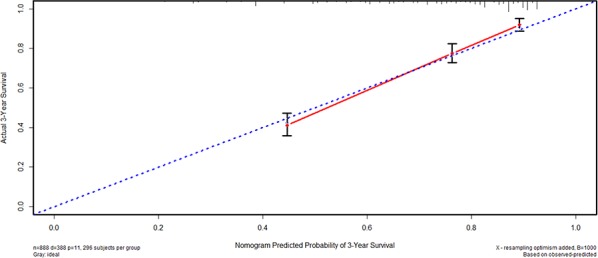
Calibration curve of the training set

**Figure 6:**
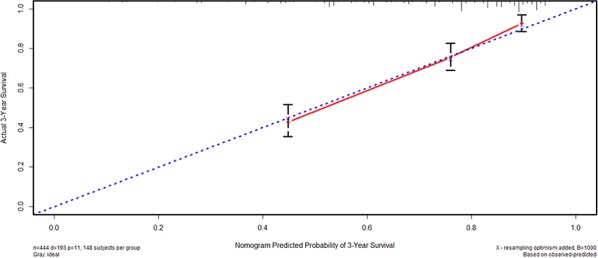
Calibration curve of the validation set

We furtherly used nomogram to predict 3-year overall survival rate of individual patient. In the training set, age, longitudinal location, tumor size, N stage, M stage, and HALP (*p* = 0.030, HR = 0.769, 95% CI 0.606–0.975) were included in the nomogram (Figure 3). Gender, age, longitudinal location, N stage, M stage, and HALP (*p* = 0.031, HR = 0.683, 95% CI 0.484–0.966) were selected in the nomogram of the validation set (Figure 4). The nomograms of two sets indicated that male, age ≥ 65, UML location, advanced N stage and M stage were the poor prognostic factors, but HHALP was still a favorable one. The results of the nomograms were similar to those of aforementioned multivariate analyses. The calibration curves of nomograms in the two sets showed that the predictive probability of 3-year survival were closely to the actual 3-year survival (Figure 5, 6).

Subsequently, we compared the predictive accuracy of prognosis between the nomogram and TNM staging system (only T stage, N stage and M stage) in the training and the validation set. The C-indexes of nomograms were 0.742 (95% CI 0.717–0.767) and 0.744 (95% CI 0.709–0.779), compared with 0.726 (95% CI 0.701–0.751) and 0.724 (95% CI 0.688–0.760) of TNM staging system in the training and the validation sets, respectively. The results indicated that the prognostic prediction accuracy of nomograms based on HALP and other parameters was significantly better than TNM staging system both in these two sets (*p* < 0.001).

## DISCUSSION

In this present study, a novel index HALP was established on the basis of the value of preoperative hemoglobin, albumin, lymphocyte and platelet, showing its potential application in the prognostic prediction of GC. Our study found that HALP was associated with many clinicopathological characteristics, like tumor size, T stage. LHALP was significantly associated with tumor progression and acted as an adverse prognostic factor in GC patients, which was confirmed both in the training and the validation sets through univariate and multivariate analyses. The nomogram also illustrated the potential value of HALP in the prognostic prediction.

The significance of HALP was the integration of these four indexes. With GC progression, many patients may manifest cancer-associated anemia, which is one of the most common paraneoplastic syndromes [13]. More importantly, GC often invades the blood vessels and causes chronic or acute stomach bleeding, which is also the main reason of anemia. Anemia may have an impact on the performance status, quality of life, clinical symptoms, tolerance and recovery of treatments like surgery and chemoradiotherapy, even prognosis [14, 15]. GC is a chronic consumption disease and albumin might be catabolized caused by cancer progression. Meanwhile, GC may lead to disorders of nutrition absorption from gastrointestinal tract. These two causes might arouse the decreasing level of albumin. Some previous studies showed that hypoalbuminemia was associated with poor prognosis of GC [16]. It has been demonstrated that with the dense intratumoral lymphocyte infiltration in early lesions, the frequencies of metastasis was reduced and the prognosis of patients was improved [17]. In contrary, the immunosuppressed individuals might have an increased risk for tumor development [18]. Many reports found that platelet was activated in GC and the plasma levels of platelet microparticles (PMP) was associated with metastasis of GC [19]. Platelet might protect cancer cells through platelet-mediated shielding effect in bloodstream [20]. Some reports showed that platelet played a role in the maintenance, growth, tumor angiogenesis, invasion, and metastasis of cancer cells through many kinds of mechanisms, like platelet-derived endothelial cell growth factor [21]. With the use of aspirin, which can inhibit the aggregation of platelet, incidence of colon cancer was reduced [22]. From the aforementioned results, we could infer that hemoglobin, albumin, and lymphocyte might be the favorable prognostic factors, but platelet might be the unfavorable one. This was why we make the definition of HALP. The results of our study also confirmed the significance of HALP, indicating that the patients with higher HALP had better prognosis than those with lower HALP.

**Figure 7:**
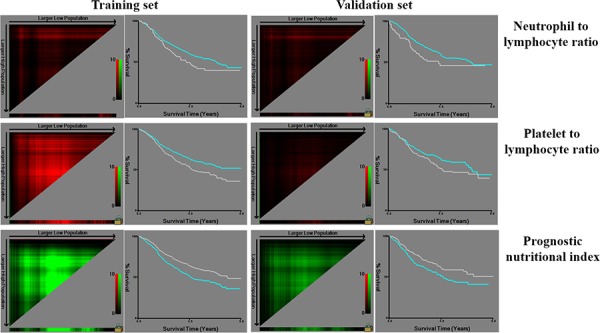
Analysis of other indexes by X-tile software

HALP was a novel index to reflect the nutritional and immune status of patients to some extent. To our best knowledge, no study had reported the significance of HALP in GC patients. Besides HALP, many other indexes, like C-reactive protein, neutrophil to lymphocyte ratio (NLR), PLR, and PNI had been widely investigated in GC patients. Some reports found that the high level of PLR was related to metastatic GC [23]. NLR was related to poor prognosis of advanced GC [11, 24]. PNI was thought as a valuable predictive indicator in the prognosis of cancer from digestive system [25, 26]. In our hospital, C-reactive protein is not the routine examination, thus, we did not choose it in this study. With respect to NLR and PLR, our study found that these two indexes were not the significant prognostic factors in the training and the validation sets through X-tile software simultaneously (Figure 7). Regarding PNI, our study revealed that PNI was the independent prognostic factor. However, we also found that PNI was significantly associated with hemoglobin (*p* < 0.001), indicating that patients with higher PNI had higher hemoglobin too. And we found the hemoglobin was also a significant prognostic factor. Therefore, we thought that the combination of PNI and hemoglobin might be more compelling than PNI alone.

In our study, to improve the reliability, the patients were randomly divided into the training set and the validation set, the baseline of which was generally comparable. The relationship between HALP and clinicopathological characteristics and the significance of HALP in prognosis were separately analyzed both in the training and the validation set, whose results were similar too. Because of the use of X-tile in the generation of the training and the validation sets, we only enrolled the patients with follow-up in this study. However, this kind of dividing in GC patients was seldom reported to explore the significance of some indexes previously.

Nomogram is a visualized and widely applied method to predict the prognosis of individual patient on the basis of some valuable parameters. In our study, we figured out the nomograms of the training set and the validation set to visually show the impact of some clinicopathological parameters on the prognosis of GC patients. According to the nomogram, the prognosis of individual patient could be well predicted. Both in the training and the validation sets, HALP was included via a stepwise algorithm and shown in nomogram. The predictive accuracy of nomogram was well illustrated through calibration curves. In the nomogram, we noticed that tumor size was included but not T stage. We thought that both tumor size and T stage were the parameters reflecting tumor development, and these two parameters might have some interactive effect when analyzed together. Our study revealed that tumor size might played a more important role than T stage in prognosis. Moreover, this study compared the predictive accuracy between nomogram and TNM staging system, and the results showed that nomogram with HALP and other parameters was better than TNM alone. However, we still thought that TNM stage were one of the most important parameter in GC, but more importantly, other indexes like HALP, tumor size should be also noticed.

The lower bound of the normal values of hemoglobin in male and female are different, with the lower bound 120 g/L in male and 110 g/L in female. In the beginning of this study, to balance this tiny difference, we added 10 g/L to the value of hemoglobin in female, however, we found that the cut-point of HALP (56.6) was almost the same with 56.8. And there was almost no changes in the constitution of patients in the training set and the validation set. Therefore, we directly used the value of hemoglobin in the calculation of HALP, irrespective of gender.

In conclusion, HALP was closely associated with clinicopathological characteristics and played a role as an independent prognostic factor of GC. Nomogram based on HALP was a good tool to accurately predict the prognosis. Preoperative calculation of HALP might be recommended as a new simple method and supplementary to predict the survival outcome of GC patients.

## MATERIALS AND METHODS

The West China Hospital research ethics committee approved retrospective analysis of anonymous data. Signed patient informed consent was waived per the committee approval, because it was a retrospective analysis.

### Patients

**Figure 8:**
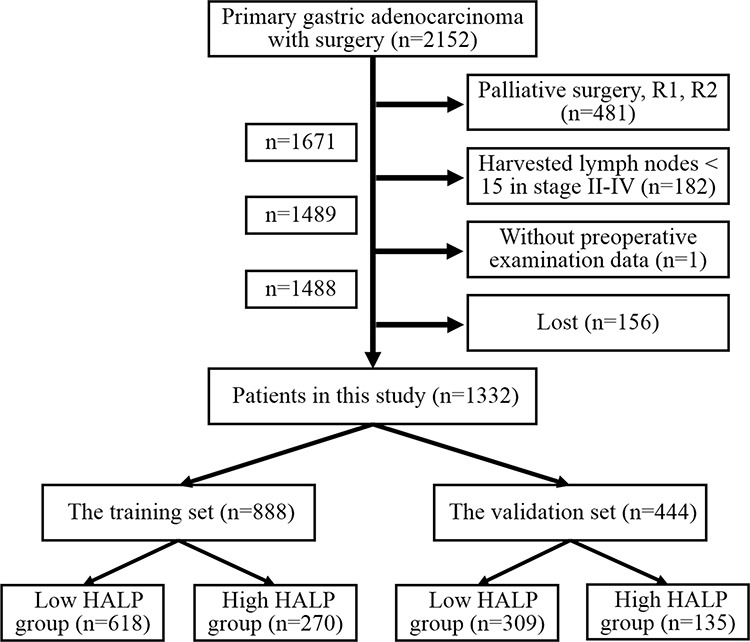
The flow chart of patients in this study

The patients, who underwent gastrectomy with curative intention for primary GC and received preoperative examinations of hemoglobin, albumin, lymphocytes, platelets in West China Hospital, Sichuan University from January 2005 to December 2011, were retrospectively included in this study. To reduce the impact of insufficient lymphadenectomy on the prognosis, we excluded the patients diagnosed with stage II-IV with less than 15 lymph nodes harvested in surgery. Finally, 1488 patients were enrolled. Among them, 1332 (89.5%) patients, who were followed up through telephones, mails and outpatient visit up to December 2014, were finally analyzed in this study (Figure 8). The clinicopathological characteristics including age, gender, tumor location, macroscopic type, differentiation grade, tumor size, vessels/nerve invasion, TNM stage according to Japanese classification of GC (3 rd English version) by JGCA [3], and follow-up information were collected.

### Definition of HALP

**Figure 9:**
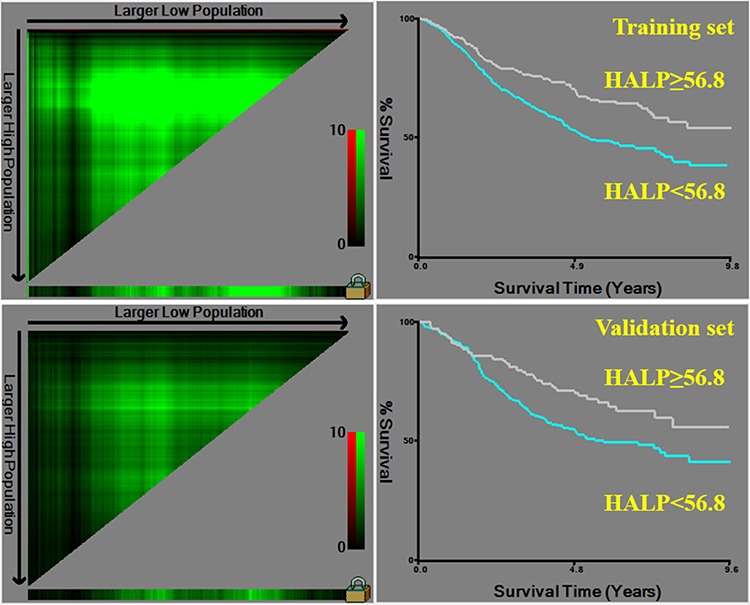
Division of patients into the training and the validation sets based on HALP by X-tile software

Preoperative hemoglobin, albumin, lymphocytes and platelets were combined to establish a new index HALP, the value of which was defined as follow: HALP = Hemoglobin (g/L) × Albumin (g/L) × Lymphocytes (/L)/Platelets (/L). With the use of X-tile software (Version 3.6.1, Yale University), 1332 patients were randomly divided into the training and the validation sets according to sample size ratio 2:1. The optimal cut-point for HALP was analyzed and calculated as 56.8 through X-tile (Figure 9). Therefore, the patients were furtherly subdivided into HALP < 56.8 (LHALP) and HALP ≥ 56.8 (HHALP) groups both in the training and the validation sets in this study.

### Statistics

Statistical analysis was performed by SPSS software (Version 22, IBM). Unordered categorical variable and ranked data was analyzed through chi-square test and rank sum test (Mann-Whitney *U* test), respectively. Student's *t*-test was used to analyze continuous data if homogeneity of variance and normal distribution. If not, rank sum test was used. Logistic regression was used in multivariate correlation analysis. Kaplan-Meier method and life-table method were used to calculate the cumulative survival rate. Log-rank test and Cox's proportional hazard regression model were conducted for univariate and multivariate survival analyses, respectively. Prism 5 for Windows (Version 5.01, GraphPad Software) was used to draft the figure of Kaplan-Meier curve. Nomogram and calibration curve were performed through R for Windows (Version 3.2.0, R Foundation for Statistical Computing) with the package of Regression Modeling Strategies (rms), in which the variables were selected according to the model by Akaike information criterion in a stepwise algorithm [27, 28]. Comparisons between the nomogram and TNM staging systems were performed with the package of Harrell Miscellaneous (Hmisc) and were evaluated by the C-index with the meaning of that the larger the C-index, the more accurate was the prognostic prediction [29]. The two-sided *p* value less than 0.05 was considered as statistical significance.
